# Pathology, Practical Medicine, and Therapeutics

**Published:** 1838-07

**Authors:** 


### PATHOLOGY, PRACTICAL MEDICINE, AND THERAPEUTICS.

### *On the Atmospheric and Epidemic Constitution of Berlin in September,* 1837.

[We give the following extract chiefly as indicative of the methodical nature of the German character, and of the attention paid in Germany to subjects comparatively neglected in this country.] Disease in this month occurred pretty frequently among the domestic animals, although not to any unusual extent. It was chiefly of a rheumatic character, frequently complicated with catarrhal and gastric affections, and more prone to become inflammatory than in the preceding months. Acute and chronic rheumatism, sore throat, rheumatic ophthalmia, inflammation of the lungs and pleuræ, inflammation of the hoofs and tendons occurred very frequently in horses. Several cases of pericarditis, peritonitis, gastritis, and enteritis preceded by rheumatic affections were also met with. Pure gastric diseases were rarely seen; colics sometimes occurred, but not so frequently as in the preceding months. Nervous diseases were rare, excepting a few cases of the staggers and a case of idiopathic tetanus. Most of the diseases were of a mild character, so that, in spite of the dangerous aspect which they in many cases assumed, few horses died. The oxen suffered chiefly from rheumatism, rheumatic and gastro-rheumatic fever, and also considerably from inflammation of the thoracic and abdominal viscera. A bull was affected with hydrophobia, about seven weeks after having been bit by a mad dog. There was little disease among the sheep, but one flock suffered considerably from inflammation of the bowels, probably from having been put to pasture on potato fields. A good many goats and some sheep suffered from indigestion, flatulence, and gastric fever. Excepting the distemper and its nervous complications there was no particular disease among the dogs, but there were a few cases of gastric disturbance, want of appetite, vomiting and diarrhœa, and one or two cases of tetters. The epidemic, alluded to in a former report, still continued in several farms among the fowls.

The prevailing diseases during the same period in the human species were cholera, dysentery, gastric fever, pulmonary inflammation, and measles.

*Medicinische Zeitung*, 1837. No. 41.

---

*Case of slow Poisoning by Oxide of Zinc.* By Dr. BUSSE, of Berlin.

A GENTLEMAN, aged forty-five, of regular habits and previously of good health, was, in 1825, attacked, without evident cause, with epilepsy, which continued to return at longer or shorter intervals. His physician recommended change of climate; and the three following years, during which there was no return of the disease, were spent in travelling, chiefly in Italy and France. But scarcely had he returned to Berlin when the fits again appeared. Various remedies were tried in vain; and the patient, happening to notice oxide of zinc, in combination with hyosciamus, recommended in Hufeland's Journal as a cure for epilepsy, resolved, without consulting any physician, to submit himself to this treatment. He secluded himself from society, and took daily, at an average, twenty grains of the oxide, till he consumed 3,246 grains, and would probably have continued his experiment to a fatal termination had not one of his relations insisted on being admitted to him, and by whom he was found in a most deplorable condition. Dr. Busse was immediately summoned, and found the patient of a pale earthy hue, wasted away, and almost idiotical: his tongue was thickly coated, the bowels were constipated, the inferior extremities cold and œdematous, the abdomen tumid, the superior extremities cold and shrivelled, and their skin dry like parchment; the pulse was about sixty, thready, and scarcely perceptible.

The oxide of zinc was immediately stopped, but not without some opposition on the part of the patient, and a purgative ordered. Light nutritive diet was allowed, with tonic and diuretic medicines; and under their use the patient rapidly improved, and soon regained his previous robust and healthy appearance, although he remained subject to epileptic attacks, which continued to recur every six or eight weeks. *Wochenschrift für die gesammte Heilkunde.* 1837. No. 19.

---

*On the Juice of the Root of the Elder-tree in Dropsies.*

THE author of this communication has employed the above juice as a hydragogue cathartic in several forms of dropsy, with the effect of completely relieving the system of the collected fluid. Its use appears to be attended with less colic than the other hydragogues with which we are acquainted; it is apt, however, to become exceedingly disgusting to the individual employing it; on which account it should be suspended for a short time, and then resumed. It should be given unmixed with other medicines. The cases which are given as illustrative of its action it is unnecessary to quote, as, from the want of very minute diagnosis, they scarcely can justify any application to other cases; but, as an addition to our hydragogues, the juice of the root of the elder-tree may occasionally be employed with advantage. It is obtained simply by subjecting the fresh root to pressure; the dose with which to commence being from two to four ounces.

*Bulletin géněral de Thérapeutique. Février*, 1836.

---

*Researches on Spontaneous Perforations of the Stomach.* By M. LEFEVRE.

THE design of the investigations pursued by M. Lefevre is, 1st, to explain the mode of formation of spontaneous perforations of the stomach; and 2d, to establish those characters which make them distinguishable from perforations the result of poisons.

It is difficult to assign the predisposing causes of this terrible accident. Nevertheless, Mr. L. thinks that persons who suffer habitually from stomach complaints are more predisposed than others. He reports six observations, all females. Can this be attributed to a modification of the form of their stomach, which Soemmering attributes to the pressure of stays? M. L. says that in all the observations which he has collected, aliments of an indigestible nature (such as peas, bacon, cabbage, &c.) have been the occasional causes of the symptoms which preceded the rupture; he therefore thinks that all substances capable of producing indigestion can equally occasion solutions of continuity of the stomach. In other words, M. L. thinks that if, by weakness or otherwise, the stomach is unable to free itself from that which embarrasses it, it is liable to rupture. This rupture has its seat naturally in the part which offers the least resistance; that is to say, in the lowest part of the organ.

The following appears to be the progress of the symptoms. Some hours after a repast, the patients are seized with a violent pain in the epigastric region with a sense of weight and swelling. To these symptoms succeed nausea and sometimes vomiting of a glairy and pituitous matter, as if it were more easy for nature to free herself from these matters than the aliments which incommode her. Little or no alvine evacuations occur, or, if they are obtained by lavements, it is evident they are the result of anterior digestions. During this period of the complaint the subjects are tormented with an extraordinary agitation: they roll upon the bed from side to side, often the belly is retracted at the middle portion whilst the epigastrium is tender and painful. The deglutition of liquids, although easy, immediately provokes nausea, and they are rejected. The tongue is pale, large, and humid. The pulse soft, regular, sometimes a little hard. However, this state of anguish is interrupted by moments of calm, although of short duration. Lastly, after a very violent attack the symptoms are aggravated and change their character. Some patients experience in the stomach the sensation of a body which displaces itself. The belly is tympanitic, and acquires an extreme sensibility. The pulse becomes frequent, small, hard, imperceptible. The skin of the extremities becomes cold and is slowly covered with a cold perspiration. The countenance expresses apprehension and terror. Finally, the patients expire with a piercing shriek which describes the immensity of their suffering. They preserve their intellectual faculties until the last moment.

*Bulletin de l'Academie Royale de Médecine. September*, 1837.

---

*On the Use of the Subcarbonate of Iron* (*Ferri Sesquioxydum,*) *in Hooping-Cough.* By Dr. Steymann.

Of all the medicines recommended in this painful and lingering disease, the subcarbonate of iron, according to Dr. Steymann, when exhibited in the second stage, is by far the best.

The following cases prove the utility of this remedy.

Case i. Henri Schrœder, eleven years old, had been suffering for nine weeks from hooping-cough, for which all the usual remedies had been exhibited, without relief. The subcarbonate of iron was administered in the dose of two grains every three hours. After the tenth dose of this remedy, all the symptoms were considerably diminished. Ten doses more, each consisting of five grains, completely subdued the disease.

Case ii. The sister of the former patient, five years old, was affected with the cough at the same period, had been treated in the same way, and with the same results. She commenced with two grains of the subcarbonate every three hours; this was increased to three grains, which completed the cure in about eight days. From the first dose the symptoms were alleviated.

Case iii. Jules Etier, five years old, was placed under the care of Dr. S. in the third week of the disease. The cough did not yield till she took the iron; and, after four days' treatment, she was completely cured. Dr. S. says he never heard the fits of coughing more violent than in this child.

Dr. S. holds out a caution against the exhibition of this remedy in the early stages, as he has found it at this period produce considerable irritation, in lieu of hastening the cure. In the first stage he employs leeches, opiates, and emetics; and, before commencing the subcarbonate, he recommends the exhibition of an emetic.

The following he has found a convenient formula: Subcarbonate of iron, twenty-five grains, white sugar, a sufficient quantity to make into ten powders; one to be taken every three hours. This dose is increased according to the age of the patient, adding a grain for every year. The effect is generally prompt; and in a few days nothing remaining but a slight catarrhal cough, which gradually disappears. *Bulletin Général de Therapeutique. Mars,* 1838.

---

*On the Nature and Treatment of Syphilis in the Province of Lithuania.*
By Dr. SCHNUHR.

SYPHILIS, in East Prussia, Lithuania, and the neighbouring provinces of Poland and Russia, is a disease so frequent, occurring amongst individuals of all ages, both old and young, that the conclusion is in a manner thrust upon the enquirer, that it is an endemic disease, capable of being communicated by other methods than by impure sexual intercourse. It is a feature of the disease in these countries, that, whilst chancres, buboes, and gonorrhœa are of comparatively rare occurrence, condylomata are met with in extraordinary abundance. The broad condylomata are much more frequent than the narrow: they are found on almost every part of the body, and may be either of primary or secondary origin. Rhagades are also extremely common, and syphilitic eruptions on the skin, swellings, and pains of the bones are also occasionally seen; but such of the last-named affections as have come under the notice of Dr. Schnuhr have always been preceded by the use of mercury.

From 1st November, 1835, to 1st November, 1837, 665 cases of syphilis came under the care of Dr. Schnuhr, viz.:

1. Broad condylomata, affecting, in the greater number of instances, the hips, anus, thighs, labia, and scrotum; and, in some cases, the back, armpits, forehead, scalp, mammæ, or angles of the mouth, occurred in 85 men, 105 women, and in 16 boys and 12 girls under fifteen years of age.—Total, 218.
2. Narrow or pointed condylomata, affecting, in men, the prepuce and glans; in women, the nymphæ and vagina, occurred in 6 men and 12 women.—Total, 18.
3. Primary ulcers of the throat and mouth occurred in 68 men, 82 women, and in 15 boys and 8 girls under fifteen years of age.—Total, 163.
4. Secondary ulcers and broad condylomata of the mouth and throat occurred in 25 men and 35 women.—Total, 60.
5. Diseases of the skin in 30 men and 21 women.—Total, 48.
6. Primary sores (chancres) on the organs of generation in 28 men and 20 women.—Total, 48.
7. Gonorrhœa and leucorrhœa in 16 men and 12 women.—Total, 28.
8. Buboes in 6 men.
9. Affections of the bones in 8 men and 16 women.—Total, 24.
10. Secondary ulcers of the extremities and various parts of the body in 18 men and 20 women.—Total, 38.
11. General syphilis in 4 men and 8 women.—Total, 12.

In the returns of preceding years, the proportion of cases of gonorrhœa and primary sores to those of condylomata is nearly the same. Condylomata were found in a great many cases, where, from minute medical examination, as well as medico-legal investigations, it appeared impossible to believe that any primary sore upon the organs of generation could have preceded their appearance. The tender age of many of the patients was in itself sufficient to rebut the idea of their being the result of sexual intercourse. Among the poorer classes, whole families sometimes occupy the same bed, and in such cases it is by no means uncommon to find all the members of the family, from the grandfather to the infant, affected with condylomata on various parts of the body.

Hospitals have been established by the Prussian government in various districts of the country for the reception of syphilitic patients. The object of these institutions is twofold; first, the immediate relief of the patient, and, secondly, the final eradication of the disease. With this latter view, each patient, on being admitted, is interrogated by the magistrate, in order, if possible, to ascertain in what manner the patient has received the contagion; but, in the generality of cases, he is unable to give any information on this head. Both man and wife may be suffering from ulcerated sore throat, and yet the organs of generation of neither will be the seat of sores or be marked with cicatrices; the older children will perhaps at the same time be found with broad condylomata round the anus, and the younger with condylomatous excrescences of the commissures of the hips and rhagades of the anus.

The peculiar features of the syphilis of Lithuania, according to Dr. Schnuhr, are, 1st, the comparatively large number of condylomata and ulcerated sore-throats; 2dly, the occurrence of these two affections as primary symptoms; 3dly, the frequent propagation of the disease by other means than by coition; 4thly, the comparatively rare occurrence of chancres; 5thly, the almost complete immunity of the lymphatic system; and, lastly, the rare occurrence of general lues, in spite of the frequent and great neglect of the primary affection.

It is supposed by Dr. Schnuhr that these peculiar features of the disease were introduced by the Russians during their occupation of the country in the seven years' war; but this idea is mere hypothesis.

Dr. Schnuhr, during the first years of his practice, employed the mercurial method of treatment; but frequent relapses induced him afterwards to abandon it for the non-mercurial plan, which he has since found to meet his most sanguine expectations. *Medicinische Zeitung.* 1837. No. 50 and 51.

---

### *On Large Doses of Tartar Emetic in the Treatment of Articular Synovial Effusions.* By Dr. Gimelle.

This is a new application of a very active medicine. Hydarthrosis is a very obstinate disease, and has frequently resisted the most energetic treatment; but can now be cured (according to Dr. Gimelle) with a surprising facility, without regard to the age, sex, or temperament of the patient, by administering, for a few days, large doses of tartar emetic.

The following cases will indicate the method of administration, and the beneficial effects produced.

Case i. Picard, a soldier, thirty-six years old, of a lymphatic temperament, and a constitution much undermined by syphilis, had his leg crushed by a fall from his horse, and was obliged to walk by the aid of a wooden leg. Fifteen days after his admission into the Hotel des Invalids, he was seized with pain in the right knee: one morning the pain became so severe that he was obliged to confine himself to his bed. In a short time there was a remarkable swelling of the knee, without change of colour, with lancinating pains upon motion: the swelling increased, and fluctuation became evident. For twenty days emollient applications were used, and antiphlogistic treatment adopted. Leeches, blisters, simple and anodyne cataplasms, were all inefficacious. Dr. G. then commenced with the tartar emetic. The first day he administered four grains; the second, six grains; the four following days, eight grains; the seventh day, ten grains; and the following day, twelve grains. It produced at first some febrile action, accompanied by vomiting and purging. From this time there was a remarkable amendment; the pain entirely ceased; the remedy was tolerated; the serous effusion was absorbed, and the movements of the articulation were made with facility. On the twelfth day the remedy was suspended, and on the fifteenth the member had recovered its normal state. Ten days after this, the left knee was attacked in a similar manner: leeches were applied which subdued in some degree the inflammation; but the pain continued acute, the volume of the knee increased rapidly, and fluctuation became apparent. The tartar emetic was at this time commenced with, in the former doses, and in a similar manner, for the first three days: it produced vomiting and purging, which then ceased, and from this period the effusion was absorbed, and the cure perfected two or three days after discontinuing the remedy. Recourse was had to small doses of opium to procure tranquil nights. There was abundant perspiration, and an abundant flow of saliva.

CASE II. Clavel, forty-seven years of age, of a lymphatic temperament, had experienced for eight or ten days, without any appreciable cause, a pain in the right elbow: at the posterior part of the elbow and upon the sides of the olecranon process, there were two round tumours, fluctuating and painful to the touch. For ten days, leeches, cataplasms, blisters, mercurial and iodated frictions were tried without benefit. The tartar emetic was then given, commencing with four grains and increasing two grains every day. It was tolerated, and the cure was complete in about ten days.

CASE III. Demolombe, a soldier, of a good constitution and sanguineous temperament, had suffered from repeated venereal attacks. The knee was of an enormous size and the fluctuation was very manifest. For four days cataplasms were applied; then thirty leeches, which alleviated the pain, but the engorgement remained stationary. Two blisters were applied, but without effect. The tartar emetic was now given, but was not tolerated for the first three days; on the fourth it was, and two days after this date the swelling was diminished four-fifths, and the cure made rapid progress. At this period, for the sake of experiment, the remedy was discontinued for two days; after the interruption the cure proceeded; it then became stationary, and then retrograded. The remedy was again administered with a similar result, and the cure perfected after taking forty-eight grains of the medicine, and never exceeding eight grains for the daily dose.

The first effect of this remedy is to calm the local pain; the second, to produce absorption of the articular effusion; and the last, to remove all traces of the results of the affection. The effect is the more prompt when the remedy is tolerated; nevertheless, in the absence of this tolerance, the disease sensibly diminishes. Dr. G. recommends, if the patient is robust, to commence by general or local bleeding.

*Bulletin Géneral de Thérapeutique Médicale et Chirurgicale. Mars,* 1838.

---

*On the Use of Strychnine.* By M. BALLY, Physician to the Hospital of La Charité.

IN addition to the violent spasmodic contractions produced by strychnine, M. Bally has observed the following symptoms of disturbance of the cerebral circulation: an appearance of stupor, vertigo, tinnitus aurium, sleeplessness, and turgescence of the capillaries of the face. The strychnine, therefore, ought never to be employed in diseases combined with, or resulting from, determination of blood to the brain. The use of the strychnine should be restricted to cases of paralysis depending on disease of the spinal marrow; and, where this part has suffered no severe injury, the greatest benefit is often derived from its employment. One remarkable case is mentioned, of a man, about fifty years of age, paralytic for five years, who was radically cured by the internal employment of strychnine. M. Bally does not strongly recommend this remedy in cases of amaurosis, on account of their frequent complication with disease of the brain. In paralysis of the extensor muscles of the hands and feet, some benefit is derived from the administration of the strychnine by the skin; but more credit is due to time than to the remedy. In cases of colica pictonum, a combination of strychnine and hydro-chlorate of morphine has been found highly successful; one-sixteenth grain of the former with one-thirty-second grain of the latter were given in the form of pill, at first twice a day and subsequently more frequently. M. Bally's object in administering this combination is—1, to alleviate pain; and, 2, "to transform the disease of the spinal marrow into another morbid affection much less severe and more easy of cure." No success attended the use of strychnine in cases of diarrhœa and dysentery, though the nux vomica is strongly recommended in those diseases by Hufeland. With regard to the dose of the strychnine, M. Bally recommends us to begin by a twentieth or one-sixteenth, and, in case the stomach is not very irritable, by one-eighth of a grain. The dose may be increased at intervals of three or four days to one, two, three, or four grains. It is rarely necessary or prudent to surpass this last dose. Two or three grains are usually sufficient to produce the desired effects. In some instances the effects produced by the strychnine are very violent; so violent, that it has been sometimes necessary to fix the patient firmly to his bed, as the strength of two persons could scarcely hold him down.

*Bulletin Génerale de Thérapeutique. February*, 1838.

---

*Neuralgia of the Head, produced by a Tumour in the Brain.*
By Professor D. Holst, of Christiania, Norway.

[In the Eighth Number of this Review we have given, from Guy's Hospital Reports, an abridged account of Dr. Bright's highly interesting "Cases and Observations illustrative of the Diagnosis where Tumours are situated in the Basis of the Brain, &c." As such cases have been hitherto very rare, we are happy to be able to communicate a similar one observed by Professor Holst, of Christiania, which was read before the Medical Society of that city, and published in the medical journal edited by Dr. Holst.*]

A lady, thirty years of age, at the end of the year 1835, began to be affected with pains in the head, soon assuming a character of great severity, apparently occupying more especially the whole galea aponeurotica capitis, but also extending downwards to the neck: the paroxysms returned at uncertain times. Many medicines were tried; amongst which, especially, quina, colchicum, and vapour-baths seemed to have beneficial effects, as the paroxysms, instead of returning several times weekly, did not recur oftener than once in four, six, or thirteen weeks. However, in the spring of 1837, after a slight catarrhal affection, they returned with the former violence. Dr. H. advised the patient to go to some warm mineral bath, such as Töplitz or Ems; but she preferred the Russian vapour-baths at Copenhagen, in which she placed much confidence. During three months' stay there, the paroxysms became complicated with atonic spasms in the muscles of the neck; she also complained of weakness of sight, not of a permanent character, nor always equally great. On her return to Christiania, she was much worse than before; the paroxysms were more frequent, more violent, and of longer duration. The pains were highly aggravated by the spasms in the muscles of the neck, by which the head was suddenly turned backwards, often to such a degree that the occiput almost touched the back, and the face was turned directly upwards. She complained now, in the intervals of paroxysms, of an obtuse but severe pain within the head, in the fore part of the brain, behind and over the right eye, occasioning the sensation as if the skull was too narrow for the brain. When walking or sitting, she kept the head fixed, as if she feared to move it, and, when lying down, she was most easy on the back. There was now also observed a permanent and complete amaurosis of the right eye, and an amaurotic amblyopia of the left likewise; and the sense of smell was so depraved that fetid things (for instance, assafœtida,) were agreeable, and fragrant scents (such as eau de Cologne) disgusting. The memory, and other mental functions, were weakened. Things remained much in the same state until the middle of December, when she was carried off by an attack of apoplexy.

The examination of the body, made thirty-two hours after death, gave the following results:—The substance of the brain was almost as soft as thick soup or gruel, especially on the right side, and most so the anterior lobe. The cortical substance was diminished in quantity, superiorly and at the sides, particularly in the right hemisphere; but at the base it was normal. There was water in all the ventricles; and, in the choroid plexus of the left lateral ventricle many hydatids. The corpora striata were soft, particularly the right, which resembled a thin jelly. The right optic thalamus had lost all natural shape, and was soft; that on the left side was also soft, but retained its form. The chiasma of the optic nerves was

* The learned author of this paper is now in England, on a royal mission to examine the prisons and prison-discipline of this country; a mission from which, we doubt not, both humanity and medical science will derive profit.

small, flat, soft; and in its neighbourhood, a large hydatid. The right optic nerve behind the chiasma was small, thin, soft; anteriorly flat, but not diminished in size. The left was flat, but not diminished; soft, but not in the same degree as the right. The olfactory nerves were also soft and atrophied. In the right anterior lobe of the cerebrum there was a tumour, which showed itself immediately on the removal of the dura mater from the lateral parts. It was surrounded on all sides by the mass of the brain, though unconnected with this except on its inferior aspect, where it received its vessels from the cerebral substance. It had a white colour, was of an irregular form, although approaching the quadrangular: it was composed of four lobes, and was so firm that incisions made in it with a knife gave a feeble strepitus: its largest circumference was $6\frac{3}{4}$ inches, its smallest $5\frac{3}{4}$; its largest diameter $2\frac{1}{2}$, its smallest $1\frac{5}{8}$, (Norway measure;) its weight 845 grains. The cerebellum was small and soft. The consistence of medulla oblongata was nearly normal.

The author explains, from these appearances, the various morbid phenomena—the amaurosis, amblyopia, and vitiated smell,—and is inclined to believe that some severe contusions of the head, which the deceased had suffered, both in childhood and a few years before her death, might have laid the first foundation of the tumour.

A case very similar to this, observed by O. Sandberg, physician in the Norwegian navy, is inserted in the same Number of *Eyr*. The patient, a man, suffered first from a severe neuralgic affection in the head: he became afterwards blind in both eyes, and died in an apoplectic attack. A tumour as large as a duck's egg was found in this case, situated between the anterior extremities of the hemispheres, and resting on the sella Turcica.

*Eyr*. 1837. Ellevte Binds, tredie Hefte.

---

*On an Epidemic Phthisis Pulmonalis.* By M. MENARD. (Read before the Academy of Medicine, 15th May, 1838.)

DURING the last four years, consumption has been so prevalent at Lunel and the neighbouring communes (in France,) that M. Menard has been led to regard it as epidemic. The disease generally begins by some visceral inflammation, chiefly of the stomach and intestines, the symptoms of which mask those of the pulmonary affection. Hæmoptysis is very rare, only having occurred thrice in thirty-three cases; and cough is very slight, particularly at the commencement. During the period of this supposed epidemic, the inflammatory affections of the chest, pleurisy, pneumonia, and pulmonary catarrh, were not observed more frequently than usual. On dissection, the ordinary tubercular state of the lungs is found, together with the changes belonging to the visceral inflammation; which, it is proper to observe, accompany the disease to its close.

*Bull. de l'Acad. Roy. de Méd.* 30 *May*, 1838.

---

*On the occurrence of several Spontaneous Fractures of the Ribs in a Scorbutic Individual.* By Dr. GOEDECHEN.

ON August 22d, 1834, a sailor was brought to the marine hospital of St. Petersburgh, who complained of extreme weakness, oppression at the chest, and a dry cough. On examination of the thorax, it appeared that, on the right side, the second, third, and fourth ribs were fractured near to their anterior extremities; and, on the left, the third and fourth were separated from their cartilaginous appendages: the abdominal extremities of the ribs and their cartilages crepitated audibly on external pressure, and formed, at intervals, angular protuberances by raising the neighbouring integument: in two places the fracture was oblique. All the fractured surfaces appeared smooth, and there was no indication of an attempt at reparation by the deposit of callus. There existed no external evidence of injury, nor could the patient recollect having sustained any hurt by fall or blow. He had not been permitted to quit his ship for a lengthened period previous to his admission into the hospital; and he, moreover, bore a good character for sobriety; which facts forbade the notion that an injury might have been received in some drunken brawl.

A careful consideration of his condition,—viz. the brownish-blue complexion, the melancholy and hectic aspect, the general wasting and want of power, with the feeble pulse and languid eye, the slow articulation, and the dry state and mottled appearance of the skin of the lower extremities,—suggested the inference, that the fractures, and separation of the ribs from their cartilages were alone referrible to a scorbutic diathesis; which conclusion was rendered more probable by the fact of the patient having been closely confined to his frigate during the summer months. Although careful bandaging was immediately had recourse to, and a wholesome diet enjoined, together with the use of acids and iron, and other active measures both internal and external, the ribs, notwithstanding, separated from their cartilages at four fresh places, and two more fractures occurred. Within two months after his admission into the hospital, the patient sunk under hectic and colliquative diarrhœa.

Examination of the body presented much general emaciation, but more especially of the lower extremities; and the integuments of the wasted abdomen were of a bluish-brown colour, dry and wrinkled; the gums were swollen and disorganized. There was nothing remarkable in the head. In the chest,—there was considerable wasting of the lungs, which were flaccid, and of a bluish-grey appearance: the heart and great vessels healthy. In the abdomen,—the omenta were deprived of fat, and the membranous viscera shrivelled; the spleen natural; the liver atrophied, shrunk, and of a carcinomatous hardness and sandy colour with speckled surface. An examination of the fractured ribs proved that the periosteum was stripped from their bodies to the extent of half an inch on either side of the seat of fracture; and that a sort of pouch had been formed around the fractured extremities of each bone, which was filled with a soft dark-red coagulum, containing small fragments of bone. The surfaces of fracture were rough but not splintered; and the neighbouring costal pleura presented no appearance of inflammation. At those spots where separation of the cartilages from the ribs had taken place, the same morbid changes had likewise occurred; the extremities of the cartilages being further softened, but neither remarkably rough nor thickened.

*Zeitschrift für die gesammte Medicin.* Band vi. Heft i.

